# Preconception care: closing the gap in the continuum of care to accelerate improvements in maternal, newborn and child health

**DOI:** 10.1186/1742-4755-11-S3-S1

**Published:** 2014-09-26

**Authors:** Sohni V Dean, Zohra S Lassi, Ayesha M Imam, Zulfiqar A Bhutta

**Affiliations:** 1 Division of Women and Child Health, Aga Khan University Karachi, Pakistan

**Keywords:** preconception, women of reproduction age, pre-pregnancy, continuum of care, adolescents

## Abstract

**Introduction:**

Preconception care includes any intervention to optimize a woman’s health before pregnancy with the aim to improve maternal, newborn and child health (MNCH) outcomes. Preconception care bridges the gap in the continuum of care, and addresses pre-pregnancy health risks and health problems that could have negative maternal and fetal consequences. It therefore has potential to further reduce global maternal and child mortality and morbidity, especially in low-income countries where the highest burden of pregnancy-related deaths and disability occurs.

**Methods:**

A systematic review and meta-analysis of the evidence was conducted to ascertain the possible impact of preconception care for adolescents, women and couples of reproductive age on MNCH outcomes. A comprehensive strategy was used to search electronic reference libraries, and both observational and clinical controlled trials were included. Cross-referencing and a separate search strategy for each preconception risk and intervention ensured wider study capture.

**Results:**

Women who received preconception care in either a healthcare center or the community showed improved outcomes, such as smoking cessation; increased use of folic acid; breastfeeding; greater odds of obtaining antenatal care; and lower rates of neonatal mortality.

**Conclusion:**

Preconception care is effective in improving pregnancy outcomes. Further studies are needed to evaluate consistency and magnitude of effect in different contexts; develop and assess new preconception interventions; and to establish guidelines for the provision of preconception care.

## Introduction

Worldwide in 2010, 287000 women died, with many more suffering long-term disability, from causes related to pregnancy and childbirth [1]. In the same year, globally 3.1 million newborn babies died in their first month of life [2], while 14.9 million were born prematurely and 2.7 million were stillborn [1]. Nearing the 2015 deadline for the Millennium Development Goals, there is a heightened awareness of this persistent burden, especially since a significant proportion of maternal, newborn and child mortality and morbidity is preventable with existing interventions.

There is widespread agreement that a continuum of care approach is necessary to further reduce maternal, newborn and child deaths [3]. At present, this continuum extends from pregnancy and childbirth, to the postnatal period (for both mothers and neonates), through early childhood. A gap remains in this continuum, particularly for adolescent girls and young women, who often receive little or no healthcare from age five until their first pregnancy. Additionally, antenatal care is too late to reduce the harmful effects that a woman’s (and her partner’s) health risks or health problems may have on the fetus during the critical period of organogenesis [4]. Preconception care completes the continuum, ensuring ongoing health surveillance and early intervention, so that women begin pregnancy in the best health possible.

Interventions that optimize women’s health before pregnancy with the intent to improve maternal and newborn health outcomes are collectively termed preconception care. The first review of the evidence in this subject area put forward this definition of preconception care: “a set of interventions that aim to identify and modify biomedical, behavioral, and social risks to a woman's health or pregnancy outcome through prevention and management” [5]. Another review suggested, “Preconception care is the entire range of measures designed to promote the health of the expectant mother and her child” [6]. Therefore to define preconception care, two essential criteria must be met: risk prevention and health promotion before pregnancy; with the aim to improve pregnancy and health outcomes for mothers and children.

To date, the evidence has typically focused on the provision of preconception care in the healthcare setting [3-6] to women or couples of reproductive age who are contemplating pregnancy or have had a previous adverse pregnancy outcome [7-11]. However, this precludes broader strategies for promoting health for all adolescents, women and men of reproductive age that could further improve outcomes for mothers and babies. In addition, the highest burden of maternal and childhood mortality and morbidity is seen in the low- and middle-income countries (LMIC) of Southeast Asia and Sub-Saharan Africa, where access to healthcare is limited, and therefore community approaches need to be developed.

The aim of this systematic review was to evaluate the effectiveness of preconception care interventions on maternal, newborn and child health (MNCH) outcomes to bridge the gap between evidence and implementation. Our objectives were to collate the data on risk factors in the preconception period and their impact on MNCH outcomes; identify research gaps; and recommend strategies for implementation.

## Framework and methods

Previous literature reviews [4,12-14] established a baseline for the conceptualization and content of preconception care. Considering the present global maternal and child health picture, and potential impact of preconception care to further accelerate improvements in outcomes, it was felt that preconception care has a broader scope and that the conceptual framework should be extended. The intention of comprehensive preconception care is to minimize health risks and optimize health for all women and couples of reproductive age [4]. Reproductive age encompasses adolescent girls age 15 and older, and women up to age 49. While the focus remains on women, it is recognized that care provided before and between pregnancies should be inclusive to adolescent boys and men, since their involvement is critical to planned and healthy pregnancies.

The literature has yet to define an exact “preconception period” and indeed there is some difficulty in doing so. We are not yet able to predict precisely when a pregnancy begins and time to conception varies for each couple. Forty one percent of pregnancies are unplanned, so pregnancy intention cannot be the basis of the preconception period [15]. Many authors describe care beginning from three months prior to pregnancy and continuing through the first trimester; however more time is required to address long-standing health problems or form positive health behaviors. We propose that the preconception period be defined as a minimum of 1-2 years prior to the initiation of any unprotected sexual intercourse that could possibly result in a pregnancy. In line with the continuum of care for MNCH, preconception care will need to overlap with care in early pregnancy, which may be referred to as “periconception care” and care provided during the postnatal period until the next pregnancy, referred to as “interconception care”.

We therefore propose that preconception care be defined as “any intervention provided to women and couples of childbearing age, regardless of pregnancy status or desire, *before* pregnancy, to improve health outcomes for women, newborns and children”. Preconception care must address the underlying and intermediate determinants of maternal and child health outcomes, such as the overall socioeconomic context and community structures and institutions, as well as the immediate biomedical and lifestyle risk factors. A complete analysis of the effects of underlying determinants and related interventions on MNCH outcomes was beyond the scope of this review, since interventions targeting literacy, women’s economic independence and other development efforts rarely link to the MNCH outcomes of interest. However, such interventions are mentioned where relevant and where it makes good sense that expanding such efforts would positively impact certain indicators of MNCH (for example methods to promote girls’ completion of school may lead to fewer adolescent pregnancies). Both delivery and demand of preconception care will need to be of a high quality and scaled up to reach target populations and achieve effects. Hence collaborative efforts will be needed in a variety of settings that aim to reach all adolescents, women and couples of reproductive age.

**Figure 1:**
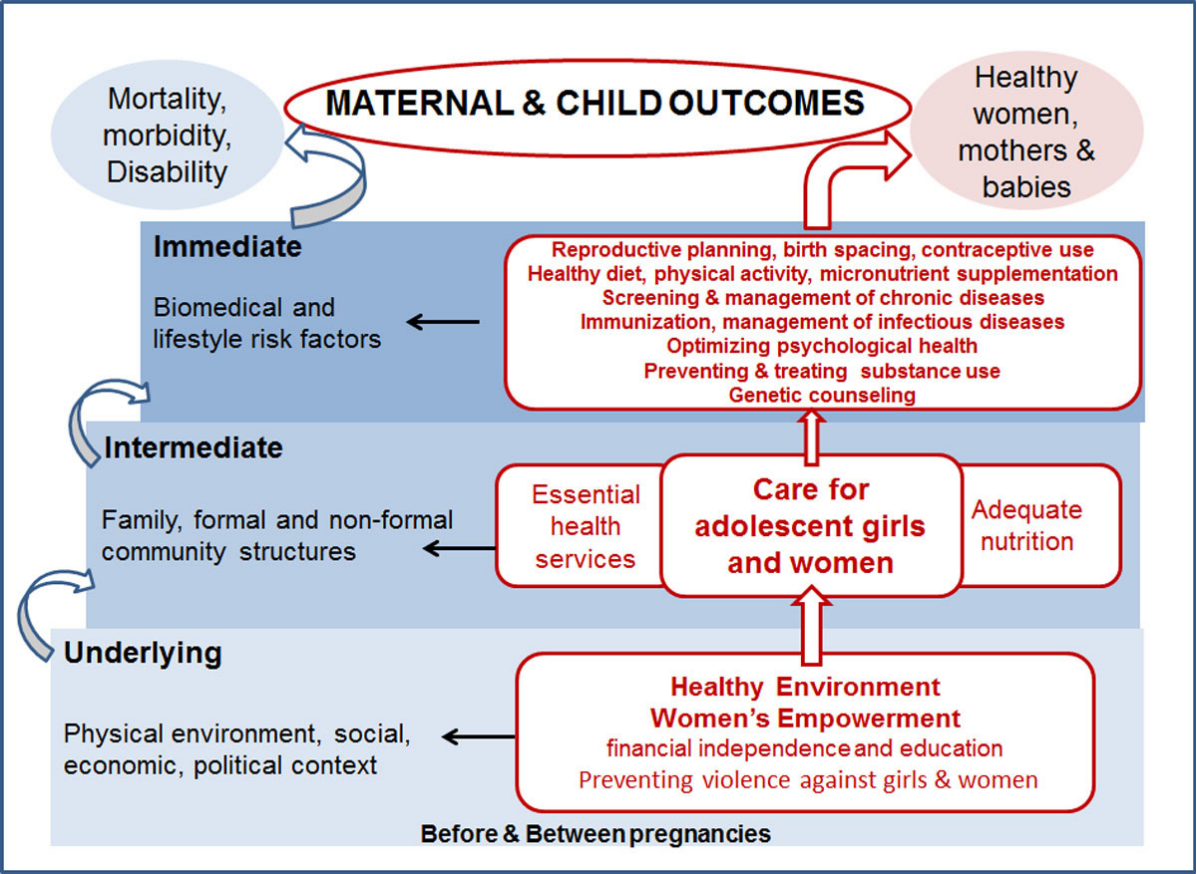
Preconception interventions framework

Within this framework (Figure 1), we developed categories of preconception risks and interventions, guided by previous reviews in the subject area. A systematic review of the evidence from all available published and unpublished papers/reports was conducted to consolidate efficacy of intervention and magnitude of risk in the preconception period. A comprehensive search strategy was employed using MeSH terms and keywords relevant to preconception care to search electronic reference libraries for global indexed and unpublished literature such as The Cochrane Library, Medline, PubMed, Popline, LILACS, CINAHL, EMBASE, World Bank's JOLIS search engine, CAB Abstracts, British Library for Development Studies BLDS at IDS, the World Health Organization (WHO) regional databases as well as the IDEAS database of unpublished working papers and Google Scholar: ("Preconception Care"[Mesh] OR “pre conception*” OR preconception* OR prepregnan* OR “pre pregnancy” OR periconception* OR “peri conception*” OR “before pregnancy” OR internatal* OR interpregnan* OR “inter pregnancy” OR interconception* OR “inter conception*” OR pregestation* OR "pre gestation*" OR pre-gestation* OR intergestation*). Although preference was given to randomized controlled trials, preconception care is a relatively new field and therefore quasi-randomized and observational studies were also included, while descriptive studies and advocacy articles helped to establish the details of a particular preconception care initiative and how preconception care has progressed since the idea was first suggested. For data to be used in the meta-analysis, the study had to specifically state that the risk or intervention occurred before pregnancy or in women of reproductive age who were not pregnant; *and* at least one outcome related to women’s, maternal, newborn or child (up to 5 years of age) health had to be reported.

Bibliographies of relevant reviews and articles were cross-referenced to ensure that no important studies were missed. Studies from organizations and experts working in the area of preconception care were sought. A second search strategy for individual preconception risks and interventions (for example, nutrition and micronutrient supplementation, infectious diseases and screening) was also performed using appropriate keywords such as: (preconception search strategy) AND (nutrition OR weight OR supplement* OR “folic acid” OR folate OR iron OR calcium OR multivitamin* OR micronutrient* OR vitamin* OR "body mass index") or for reproductive planning AND ((contraception OR “family planning” OR “pregnancy planning” OR “reproductive planning” OR “child spacing” OR “birth spacing” OR “birth intervals” OR “pregnancy spacing” OR “interpregnancy interval” OR “preventing pregnancy” OR “pregnancy prevention” OR “pregnancy in adolescence” OR “teen* pregnancy” OR “unwanted pregnancy” OR “unintended pregnancy” OR “unplanned pregnancy”)).

Titles and abstracts were screened, and data extracted independently by two study researchers, and thequality of each study was assessed using respective criteria such as Cochrane criteria for randomized and quasi randomized studies [16], and STROBE (Strengthening the Reporting of Observational studies in Epidemiology) [17] for observational studies. Meta-analyses of quantitative studies were conducted where possible using Review Manager (RevMan) software Version 5.1. For dichotomous data, the results were presented as summary risk ratio (RR)/odds ratio (OR) (as quoted in individual studies) with 95% confidence intervals (CI) and for continuous data, mean difference (MD) were used between trials if outcomes are measured comparably. For analyzing and pooling cluster randomized trial data, the entire cluster was used as the unit of randomization and the analysis was adjusted for design [18]. The data of cluster-randomized trials were incorporated using generic inverse variance (GIV) method in which logarithms of RR estimates were used along with the standard error of the logarithms of risk ratio estimates. The level of attrition was noted for each study and its impact on the overall assessment of treatment effect was explored by using sensitivity analysis. Heterogeneity between trials was assessed using the I-squared statistic, P value of <0.1 (on chi-square) and by visual inspection of forest plots. When high levels of heterogeneity between trials (exceeding 50%) was identified, further exploration was conducted by subgroup analysis. We initially undertook fixed-effects meta-analysis for combining data where trials examined the same intervention, but then repeated the analysis and applied random-effects meta-analysis as an overall summary because of substantial methodological heterogeneity between and among the studies. The differences in estimates from two sub-group meta-analysis were tested using the method described by Altman and Bland [19]. Some disaggregated analysis (quantitative) were performed on interventions delivered alone versus delivered in combinations, preventive versus therapeutic, interventions involving community mobilization and those delivered in community setup versus those in facility setup. The findings were shared at international meetings with experts in the field of maternal and child health, which gave greater strength to the results. An exercise to systematically and transparently set research priorities for preconception care in LMICs with the greatest burden of maternal and child mortality and morbidity over the next decade was also undertaken [20].

## Results

This first paper in the series presents only the results of general preconception counseling, specific risk factors and interventions are further discussed in subsequent papers [21-25]. It has been shown that women who receive preconception care and counseling are likely to develop positive health behaviors, such as daily pre-pregnancy multivitamin consumption, early entry into prenatal care, and cessation of alcohol use [26]. We identified 19 randomized controlled trials of preconception counseling [14,27-45].

A contrast was observed with studies undertaken in higher income contexts including the USA, Netherlands, Australia, and Hungary utilizing healthcare facilities; whereas the studies carried out in Nepal, Bangladesh, Pakistan, India, and Bolivia developed community groups. The studies in health care facilities had trained providers focusing on individual counseling and care for women or couples up to one year before conception. Healthcare providers were able to reach women who already attended clinics more easily than through health records. Even though most women have at least one risk factor, and many had multiple risks, they did not report anxiety after receiving preconception counseling.

**Figure 2:**
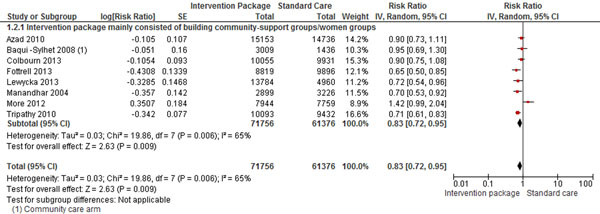
Preconception community counselling effect on neonatal mortality: evidence from controlled trials

**Figure 3:**
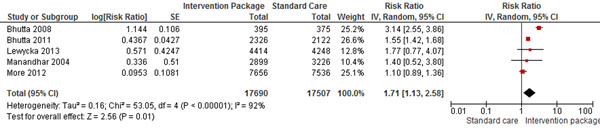
Preconception community counseling effect on breastfeeding: evidence from controlled trials

Community-based studies mainly focused on educating women about pregnancy and child care by building women support groups in the community where all the women of reproductive age were encouraged to participate. Initially, a community health worker would teach a small group of women, and then they would go out to educate other women, and rarely husbands, in their own communities. The pooling of these interventions identified that preconception care and counseling improved women’s health behaviors- women were 3 times more likely to quit smoking in one study [32],; women were five to six times more likely to use folic acid if they had received counseling in a health facility; women were also 71% more likely to breastfeed their newborn, 82% more likely to use safe delivery kits at home births in developing countries, and 39% more likely to obtain antenatal care. With early and increased contact with health professionals and increased awareness from routine checkups, MNCH outcomes also showed significant improvement- women were less likely to have an ectopic pregnancy or miscarriage; had lower rates of STIs; and were more likely to identify their pregnancy as intended. The community based studies showed that in developing countries, education on pregnancy and childbirth for women during preconception show promise as a means to improve maternal and child health. Promoting health before conception can increase antenatal care seeking by 39%, reduce neonatal mortality by 17% (RR 0.83; 95% CI: 0.72 to 0.95) (Figure 2), increase the use of safe delivery kits at home births in developing countries by 1.82 times (RR 1.82; 95% CI: 1.10 to 3.02), and increase the likelihood of breastfeeding by 71% (RR 1.71; 955 CI: 1.13 to 2.58) (figure 3).

## Discussion

Each year 16 million adolescent girls between the ages of 15 and 19 give birth. According a survey done in 2011 among U.S. High school students, 47% have ever had intercourse, of which 40% did not use condom [46]. Adolescents consistently demonstrate lower levels of contraceptive use during sexual activity than their adult counterparts and approximately one in five adolescent girls report having experienced sexual coercion [47]. Adolescent girls are therefore especially vulnerable to sexually-transmitted infections and unintended pregnancies. Their odds of dying from pregnancy-related causes are doubled and their babies are more likely to be preterm and low birth weight [48] than women aged 20-29. Approximately 222 million women (in LMICs only) have an unmet needs for contraception, putting them at risk for unplanned pregnancies, multiple closely-spaced pregnancies and unsafe abortions [49]. Many women enter pregnancy with depleted nutritional reserves, being underweight, having micronutrient deficiencies, or both [50]. For example, folic acid is known to be protective against neural tube defects in newborns, however in both high income countries (HICs) and LMICs only one third of women consume a folic acid supplement [51]. Obesity is a growing problem and 300 million women are considered to be overweight or obese according to their body mass index [52], which puts them in danger of gestational diabetes [53] and hypertension [54,55]; increases their need for obstetric intervention; and negatively affects neonatal outcomes[56,57]. Women who have chronic health problems ranging from diabetes to anemia and mental health disorders, especially depression, also have worse pregnancy outcomes for themselves and their newborns. Preconception care is therefore important from a public health perspective since it could reduce health risks and improve global outcomes for women, mothers and babies.

Preconception care is perhaps most needed in LMIC of Sub-Saharan Africa and South Asia, where the overwhelming majority of maternal and child deaths continue to occur [57,58]. The state of maternal and child health in LMIC is reflective of the underlying determinants of health in these contexts, such as poverty, lack of water and sanitation facilities, and food shortages. Weak education and health systems, as well as sociocultural norms that perpetuate gender inequality, mean that women are often not empowered to gain access to healthcare or make decisions about their own wellbeing [59]. Providing comprehensive care before pregnancy to adolescent girls and women in these resource-poor regions, especially interventions at the community and primary care levels, will have benefits for the health of women, children and families overall. Better health for women and children in LMICs will reduce disparities and increase equity in global health and development.

We identified some good quality randomized controlled trials with significantly different contexts and processes that demonstrate the potential of preconception care to improve MNCH outcomes. Given the variable parameters, timeframes and targeted groups that have been used in preconception care research, it is not surprising that the MNCH outcomes reported are not always consistent across studies and therefore limit pooled analysis. The framework used in this review attempts to broaden the scope of preconception care. Challenges also remain in preconception care implementation. The groups at highest risk for adverse MNCH outcomes are difficult to reach through traditional healthcare avenues. Preconception care may be seen as stigmatizing if it is only associated with sexual and reproductive health. Some women and couples may not desire pregnancy and others are unable to become pregnant; they may also choose to delay pregnancy or are simply not considering it at the present time- although preconception care may be targeted to these audiences they may not be receptive to it. Health systems and community-based strategies will have to be strengthened to provide preconception care.

## Conclusion

In recent years, as support for improving women’s and children’s wellness through preconception care has grown in the public health domain, and with increasing concern over the need to provide such care equitably, new strategies are being tested for delivery and integration within and outside the healthcare system. It has been shown that women who receive preconception counseling are likely to develop positive health behaviors; therefore preconception care may be an effective means to improve pregnancy outcomes. However, further studies are needed to evaluate consistency and magnitude of effect in different contexts; develop and assess new preconception interventions; and to establish guidelines for the provision of preconception care. This paper is the first in the series and underscores the scope and possibility of preconception care to improve the health of women, mothers and young children within the expanded framework described. This series takes the agenda for preconception care a step beyond the content of care. In the following Papers [21-25], we evaluate the effectiveness of preconception interventions in the domains of promoting adolescent health; reproductive planning; improving nutritional status; screening and treatment of infectious diseases; diagnosis and management of non-communicable diseases; promoting psychological health; and preventing harmful environmental exposures. In concluding papers [60,61], we emphasize the next steps: strategies to maximize delivery of effective interventions, the need to develop innovative ways to detect risk and institute early measures to optimize health in the preconception period. At present, all healthcare providers for adolescent girls and women of reproductive age should recognize the importance of the continuum of care even before pregnancy, to improve the health of mothers and children. They can and should begin to provide preconception care to every woman every time simply by asking if they wish to become pregnant or could become pregnant.

## Competing interests

We do not have any financial or non-financial competing interests for this review.

## Peer review

Peer review reports are included in additional file 1.

## Supplementary Material

Additional file 1 Peer review reports. Click here for file

## Declarations

This article has been published as part of *Reproductive Health* Volume 11 Supplement 2, 2014: Preconception interventions. The full contents of the supplement are available online at http://www.reproductive-health-journal.com/supplements/11/S3. Publication charges for this collection were funded by the Partnership for Maternal, Newborn & Child Health (PMNCH).
