# Supplementary material for: Preconception care: closing the gap in the continuum of care to accelerate improvements in maternal, newborn and child health
Source: Reprod Health. 2014 Sep 26;11(Suppl 3):S1. doi: 10.1186/1742-4755-11-S3-S1 (PMC4196556; doi:10.1186/1742-4755-11-S3-S1)
Supplement: Additional file 1 — Peer review reports. [file 1742-4755-11-S3-S1-S1.pdf]

Referee's comments to the authors– this sheet WILL be seen by the author(s) and published with the article

Title	**Preconception Care: Closing the gap in the continuum of care to accelerate improvements in maternal, newborn and child health**
Author(s)	Sohni V Dean, Zohra S Lassi, Ayesha M Imam, Zulfiqar A Bhutta
Referee's name	Geordan Shannon

When assessing the work, please consider the following points, where applicable:

1. Is the question posed by the authors new and well defined?
2. Are the methods appropriate and well described, and are sufficient details provided to replicate the work?
3. Are the data sound and well controlled?
4. Does the manuscript adhere to the relevant standards for reporting and data deposition?
5. Are the discussion and conclusions well balanced and adequately supported by the data?
6. Do the title and abstract accurately convey what has been found?
7. Is the writing acceptable?

Please make your report as constructive and detailed as possible in your comments so that authors have the opportunity to overcome any serious deficiencies that you find and please also divide your comments into the following categories:

- Major Compulsory Revisions (which the author must respond to before a decision on publication can be reached)
- Minor Essential Revisions (such as missing labels on figures, or the wrong use of a term, which the author can be trusted to correct)
- Discretionary Revisions (which are recommendations for improvement but which the author can choose to ignore)

Where possible please supply references to substantiate your comments.

When referring to the manuscript please provide specific page and paragraph citations where appropriate.

General comments:

Thank you for the opportunity to review the journal article titled: *Preconception Care: Closing the gap in the continuum of care to accelerate improvements in maternal, newborn and child health*. I believe this article addresses an incredibly important area of maternal and child health care in developing countries and also introduces a new framework for clarifying preconception care. Indeed closing the gap in the continuum of care may lead to closing the gap in many important health inequalities.

Major compulsory revisions:

1. Background section:
   Thank you for providing such a detailed and interesting background to the work. It almost seems like a review in and of itself! The only downside to this (very important information) is the detraction from the SR and meta-analysis itself. I understand that a lot of this information is important background, but is there any way to restructure at least parts of it into a discussion instead?

(continue on the next sheet)

Continued:

1. Framework and Methods section
   The proposed framework is very interesting and addresses the very difficult and broad concept of preconception care and the social determinants of health. However, I would like to see more information in the methodology. Namely:
   a. What standardized criteria did you use to assess the quality of each study?
   b. Of the quantitative studies, some report odds ratios and some relative risks. How did you account for such differences given the spread of data over randomized, pseudo-randomized and observational studies?
   c. What data from international organizations was sought and how did this get used in the analysis if at all?
   d. The actual details of the meta-analysis itself are lacking and it would be fantastic to see some more information about how you grouped different studies into certain subgroups and also if you performed any subgroup analysis. And what do the subgroups mean?

3. Results section

The results section reads more like a discussion to me. The first paragraph states some results but then subsequent paragraphs are much better suited to a discussion. At the beginning of the results section, key information about the SR and meta-analysis is missing. How many studies were included? What are they on? What groups naturally formed? What areas have more information than others? What areas have no information? Pooled analysis in the meta-analysis should then be discussed. What were the pooled effects and in what categories? Is this significant?
Figure 2 is unfortunately missing.
Both Figures could be referred to more in the body of the text as this is the key result.

Minor essential revisions:

1. Paragraph 1, "what is preconception care," last sentence: delete "it is clear that" and there seems to be three elements here not two? 1) risk prevention, 2) health promotion, and 3) to improve pregnancy outcomes…
2. Paragraph 2, "what is preconception care," second sentence: commas not semi-colons.
3. Paragraph 3, "why is preconception care needed?" second sentence: should reference be put at the end or should another reference be used to support the statement that "1 in 5 girls report having experienced sexual coercion"?
4. Paragraph 4, "why is preconception care needed?" third sentence: please reference
5. Paragraph 2, "who should receive preconception care and when should it begin?" third sentence: please reference
6. Throughout: numbers smaller than ten can be written as a word. If they're larger than this they are written as a number. Numbers at the start of a sentence should be written as a word, eg 40% should be forty percent (as it is at the start of one of your sentences)
7. Paragraph 3 of the results section is very bulky – is it possible to break this up into two smaller section? Also, this seems more like a discussion, as I mentioned above

Referee's comments to the authors– this sheet WILL be seen by the author(s) and published with the article

Title	**Preconception Care: Closing the gap in the continuum of care to accelerate improvements in maternal, newborn and child health**
Author(s)	Sohni V Dean, Zohra S Lassi, Ayesha M Imam, Zulfiqar A Bhutta
Referee's name	Ana Langer

When assessing the work, please consider the following points, where applicable:

1. Is the question posed by the authors new and well defined?
2. Are the methods appropriate and well described, and are sufficient details provided to replicate the work?
3. Are the data sound and well controlled?
4. Does the manuscript adhere to the relevant standards for reporting and data deposition?
5. Are the discussion and conclusions well balanced and adequately supported by the data?
6. Do the title and abstract accurately convey what has been found?
7. Is the writing acceptable?

Please make your report as constructive and detailed as possible in your comments so that authors have the opportunity to overcome any serious deficiencies that you find and please also divide your comments into the following categories:

- Major Compulsory Revisions (which the author must respond to before a decision on publication can be reached)
- Minor Essential Revisions (such as missing labels on figures, or the wrong use of a term, which the author can be trusted to correct)
- Discretionary Revisions (which are recommendations for improvement but which the author can choose to ignore)

Where possible please supply references to substantiate your comments.

When referring to the manuscript please provide specific page and paragraph citations where appropriate.

General comments: Very good paper on a critical but neglected issue. The research questions are well defined, the methods are appropriate and adequately described, and the data are clearly presented. The abstract accurately represents the paper, and the article is very well written. The authors propose a good and somehow original definition of poorly define periods, i.e. "preconception" and "periconception."The "preconception interventions framework" incorporates a comprehensive list of relevant factors that may affect the health of mothers, fetuses, newborns and children.

Major compulsory revisions:

- There is a certain disconnect between elements described as critical in certain sections of the paper and the data that the authors described and analyzed. For instance, after clearly explaining why contraception is a critical element of periconceptional care, no data on contraception are included in the search and results section, and contraception is not mentioned in the short discussion. Along the same lines, the very comprehensive conceptual framework includes critical intermediate and underlying factors, but the authors don't analyze their effects on maternal, perinatal and child health. In fact, they limit their focus to the immediate factors. Probably, analyzing the effects of social determinants and contextual factors on maternal, perinatal and child health was beyond the scope of this project. If that is the case, I recommend that the authors make that explicit in the discussion.
- The discussion ("conclusion") is too short and superficial. I recommend that the authors expand the very good points they make and add some discussion about the practical difficulties that projects and programs targeting adolescents and other vulnerable groups face when trying to reach them (e.g. adolescents are generally healthy and don't use the health system; there is stigma associated with sexual and reproductive health; adolescents who are not enrolled in schools and married adolescents are particularly challenging to reach, etc.), and the difficulties associated with identifying and including women "soon to be pregnant" in research projects and programs.

Minor essential revisions:

(continu

- Figure 2 is not included in the paper.
- The authors do not provide an explanation about the factors that may explain some of the benefits of preconceptional care that they have identified, e.g. reduced likelihood of having an ectopic pregnancy (is it because STIs have lower rates in intervention groups?) or miscarriages (first paragraph of the results section).
- The short reference to an increased interest in preconception care and new strategies being tested is not clear or supported by any references. Similarly, the authors mention one study that involves home visits, but don't explain what is the relationship between that study and the increased attention on preconception care and don't provide a reference (second paragraph of the results section).
- The description of recurrent pregnancy loss and what can explain it and done about it (third paragraph of the results section) is far too long and detailed, particularly considering the whole length of the paper and the level of analysis of other outcomes (which is, basically, nil). I recommend that the authors significantly reduce the length of that section and analyze a few other factors that may explain other critical outcomes and what can be done to modify them favorably.

Comments from the Supplement Editor

In addition to the above comments, please improve the design of your manuscript. An introduction is needed, putting the article in the context of other articles in the supplement, the objectives of this article must be stated and a discussion section is needed.

Referee's comments to the authors– this sheet WILL be seen by the author(s) and published with the article

Title	**Preconception Care: Closing the gap in the continuum of care to accelerate improvements in maternal, newborn and child health**
Author(s)	Sohni V Dean, Zohra S Lassi, Ayesha M Imam, Zulfiqar A Bhutta
Referee's name	Geordan Shannon

When assessing the work, please consider the following points, where applicable:

1. Is the question posed by the authors new and well defined?
2. Are the methods appropriate and well described, and are sufficient details provided to replicate the work?
3. Are the data sound and well controlled?
4. Does the manuscript adhere to the relevant standards for reporting and data deposition?
5. Are the discussion and conclusions well balanced and adequately supported by the data?
6. Do the title and abstract accurately convey what has been found?
7. Is the writing acceptable?

Please make your report as constructive and detailed as possible in your comments so that authors have the opportunity to overcome any serious deficiencies that you find and please also divide your comments into the following categories:

- Major Compulsory Revisions (which the author must respond to before a decision on publication can be reached)
- Minor Essential Revisions (such as missing labels on figures, or the wrong use of a term, which the author can be trusted to correct)
- Discretionary Revisions (which are recommendations for improvement but which the author can choose to ignore)

Where possible please supply references to substantiate your comments.

When referring to the manuscript please provide specific page and paragraph citations where appropriate.

General comments:

Thank you for the opportunity to review the journal article titled: *Preconception Care: Closing the gap in the continuum of care to accelerate improvements in maternal, newborn and child health*. I believe this article addresses an incredibly important area of maternal and child health care in developing countries and also introduces a new framework for clarifying preconception care. Indeed closing the gap in the continuum of care may lead to closing the gap in many important health inequalities.

Thanks.

Major compulsory revisions:

2. Background section:
   Thank you for providing such a detailed and interesting background to the work. It almost seems like a review in and of itself! The only downside to this (very important information) is the detraction from the SR and meta-analysis itself. I understand that a lot of this information is important background, but is there any way to restructure at least parts of it into a discussion instead?
   Yes, we have restructured it now.

Discretionary revisios:

2. Framework and Methods section

The proposed framework is very interesting and addresses the very difficult and broad concept of preconception care and the social determinants of health. However, I would like to see more information in the methodology. Namely:

e. What standardized criteria did you use to assess the quality of each study?
   Added in 6th paragraph under the heading of "Framework and Methods for the systematic

f. Of the quantitative studies, some report odds ratios and some relative risks. How did you account for such differences given the spread of data over randomized, pseudo-randomized and observational studies?

Added in 6th paragraph under the heading of "Framework and Methods for the systematic review"

"For dichotomous data, the results were presented as summary risk ratio (RR)/odds ratio (OR) (as quoted in individual studies) with 95% confidence intervals (CI) and for continuous data, mean difference (MD) were used between trials if outcomes are measured comparably."

g. What data from international organizations was sought and how did this get used in the analysis if at all?

Studies and not the Data. Corrected now. Those organization pin pointed the studies that we missed to include in our review.

h. The actual details of the meta-analysis itself are lacking and it would be fantastic to see some more information about how you grouped different studies into certain subgroups and also if you performed any subgroup analysis. And what do the subgroups mean?

Added in the 6th paragraph under the heading of "Framework and Methods for the systematic review"

"Some disaggregated analysis (quantitative) were performed on interventions delivered alone versus delivered in combinations, preventive versus therapeutic, interventions involving community mobilization and those delivered in community setup versus those in facility setup."

4. Results section

The results section reads more like a discussion to me. The first paragraph states some results but then subsequent paragraphs are much better suited to a discussion. At the beginning of the results section, key information about the SR and meta-analysis is missing. We have added that in detail in methods section. How many studies were included? Added in first paragraph of results. What are they on? What groups naturally formed? What areas have more information than others? What areas have no information? Pooled analysis in the meta-analysis should then be discussed. What were the pooled effects and in what categories? Is this significant? We have modified and answered all the queires in the paragraphs under results.

Figure 2 is unfortunately missing.

Added

Both Figures could be referred to more in the body of the text as this is the key result.

Added under the heading of results.

Minor essential revisions:

8. Paragraph 1, "what is preconception care," last sentence: delete "it is clear that" and there seems to be three elements here not two? 1) risk prevention, 2) health promotion, and 3) to improve pregnancy outcomes…

Deleted the suggested.

Also clarified the two elements as "Therefore to define preconception care, two essential criteria must be met: risk prevention and health promotion before pregnancy to improve pregnancy outcomes for mothers and children."

9. Paragraph 2, "what is preconception care," second sentence: commas not semi-colons.

Revised the whole section

10. Paragraph 3, "why is preconception care needed?" second sentence: should reference be put at the end or should another reference be used to support the statement that "1 in 5 girls report having experienced sexual coercion"?

Dragged the reference to the end. Also added another reference with another sentence to it "According a survey done in 2011 among U.S. High school students, 47% ever had intercourse, of which 40% did not use condom". We have also moved this paragraph to the discussion at the end.

11. Paragraph 4, "why is preconception care needed?" third sentence: please reference

Added

12. Paragraph 2, "who should receive preconception care and when should it begin?" third sentence: please reference added and moved to discussion section at the end.

13. Throughout: numbers smaller than ten can be written as a word. If they're larger than this they are written as a number. Numbers at the start of a sentence should be written as a word, eg 40% should be forty percent (as it is at the start of one of your sentences)

Corrected and moved to the second paragraph of the methods section.

14. Paragraph 3 of the results section is very bulky – is it possible to break this up into two smaller section? Also, this seems more like a discussion, as I mentioned above

Yes. Now divided into two parts.

Referee's comments to the authors– this sheet WILL be seen by the author(s) and published with the article

Title	**Preconception Care: Closing the gap in the continuum of care to accelerate improvements in maternal, newborn and child health**
Author(s)	Sohni V Dean, Zohra S Lassi, Ayesha M Imam, Zulfiqar A Bhutta
Referee's name	Ana Langer

When assessing the work, please consider the following points, where applicable:

1. Is the question posed by the authors new and well defined?
2. Are the methods appropriate and well described, and are sufficient details provided to replicate the work?
3. Are the data sound and well controlled?
4. Does the manuscript adhere to the relevant standards for reporting and data deposition?
5. Are the discussion and conclusions well balanced and adequately supported by the data?
6. Do the title and abstract accurately convey what has been found?
7. Is the writing acceptable?

Please make your report as constructive and detailed as possible in your comments so that authors have the opportunity to overcome any serious deficiencies that you find and please also divide your comments into the following categories:

- Major Compulsory Revisions (which the author must respond to before a decision on publication can be reached)
- Minor Essential Revisions (such as missing labels on figures, or the wrong use of a term, which the author can be trusted to correct)
- Discretionary Revisions (which are recommendations for improvement but which the author can choose to ignore)

Where possible please supply references to substantiate your comments.

When referring to the manuscript please provide specific page and paragraph citations where appropriate.

General comments: Very good paper on a critical but neglected issue. The research questions are well defined, the methods are appropriate and adequately described, and the data are clearly presented. The abstract accurately represents the paper, and the article is very well written. The authors propose a good and somehow original definition of poorly define periods, i.e. "preconception" and "periconception."The "preconception interventions framework" incorporates a comprehensive list of relevant factors that may affect the health of mothers, fetuses, newborns and children.
Thanks

Major compulsory revisions:

- There is a certain disconnect between elements described as critical in certain sections of the paper and the data that the authors described and analyzed. For instance, after clearly explaining why contraception is a critical element of periconceptional care, no data on contraception are included in the search and results section, and contraception is not mentioned in the short discussion. Contraception is included in the search. Please refer to third paragraph, 7 line under the heading framework and methods for the systematic review. The detailed section on contraception and family planning is a part of Paper 2 of this series. Along the same lines, the very comprehensive conceptual framework includes critical intermediate and underlying factors, but the authors don't analyze their effects on maternal, perinatal and child health. In fact, they limit their focus to the immediate factors. Probably, analyzing the effects of social determinants and contextual factors on maternal, perinatal and child health was beyond the scope of this project. If that is the case, I recommend that the authors make that explicit in the discussion. Added this in the third paragraph under the heading of framework and methods of the systematic review.
- The discussion ("conclusion") is too short and superficial. I recommend that the authors expand the very good points they make and add some discussion about the practical difficulties that projects and programs targeting adolescents and other vulnerable groups face when trying to reach them (e.g. adolescents are generally healthy and don't use the health system; there is stigma associated with sexual and reproductive health; adolescents who are not enrolled in schools and married adolescents are particularly challenging to reach, etc.), all the suggestion in there.

(continu

and the difficulties associated with identifying and including women "soon to be pregnant" in research projects and programs. We now removed some of the sections from the results which were very relevant to the discussion now under the heading of discussion. We have tried and addressed

Minor essential revisions:
Discretionary revisions:

- Figure 2 is not included in the paper.
  Included
- The authors do not provide an explanation about the factors that may explain some of the benefits of preconceptional care that they have identified, e.g. reduced likelihood of having an ectopic pregnancy (is it because STIs have lower rates in intervention groups?) or miscarriages (first paragraph of the results section).
  Added in the first paragraph under the results section "With early and increased contact with health professionals and increased awareness from routine checkups,…"
- The short reference to an increased interest in preconception care and new strategies being tested is not clear or supported by any references. Similarly, the authors mention one study that involves home visits, but don't explain what is the relationship between that study and the increased attention on preconception care and don't provide a reference (second paragraph of the results section).
  That was out of context and therefore removed.
- The description of recurrent pregnancy loss and what can explain it and done about it (third paragraph of the results section) is far too long and detailed, particularly considering the whole length of the paper and the level of analysis of other outcomes (which is, basically, nil). I recommend that the authors significantly reduce the length of that section and analyze a few other factors that may explain other critical outcomes and what can be done to modify them favorably.
  We have now reduced the length and added other contents that readers will be reading in other papers of the series.
